# OA01.04. The effectiveness and cost effectiveness of acupressure for chemotherapy-related nausea

**DOI:** 10.1186/1472-6882-12-S1-O4

**Published:** 2012-06-12

**Authors:** J Hughes, A Molassiotis, W Russell, M Breckons, M Lloyd-Williams, J Richardson

**Affiliations:** 1 Royal London Hospital for Integrated Medicine, London, United Kingdom; 2 University of Manchester, Manchester, United Kingdom; 3 Plymouth University, Plymouth, United Kingdom; 4 University of Liverpool, Liverpool, United Kingdom

## Purpose

To assess the clinical effectiveness and cost effectiveness of self-acupressure using wristbands in addition to standard care in the management of chemotherapy-induced nausea.

## Methods

Randomised three-group sham-controlled trial. Patients with heterogeneous cancer diagnoses receiving low, moderate and highly emetogenic chemotherapy randomised to receive, in addition to standardised antiemetics, either acupressure wristbands, sham acupressure wristbands or antiemetics alone. Patients were instructed to wear the wristbands for 7 days during each cycle of chemotherapy. Patients participated for 4 cycles of chemotherapy. An economic evaluation was carried out based on drug and health service utilisation. A nested qualitative interview study was also incorporated to shed more light into quantitative findings.

## Results

500 patients randomised in the three study groups. Primary outcome analysis (nausea in cycle (1) revealed no statistically significant differences between the three groups. When the two wristband groups were examined together against antiemetics only, statistical level almost reached significance (p=0.07). No significant differences were detected in relation to vomiting outcomes, anxiety, and quality of life measures. The cost-effectiveness evaluation revealed that both real and sham wristbands were associated with reduced costs compared to the antiemetics group only (p<0.001). The qualitative data suggested that patients perceived the wristbands (both real and sham) as effective and helpful to manage their nausea experience. Minor and transient side effects from the use of the wristbands were observed.

## Conclusion

No clear recommendations can be made about the use of acupressure wristbands in the management of chemotherapy-related nausea, as results did not reach statistical significance. However, the use of wristbands was associated with significant health care cost-savings, they were safe and perceived to be effective by patients. Before rejecting this intervention, we need to consider the therapeutic effects of placebos in situations such as the management of nausea with a low-cost and safe intervention that may enhance the effect of antiemetic drugs.

